# Role of chemical crosslinking in material-driven assembly of fibronectin (nano)networks: 2D surfaces and 3D scaffolds

**DOI:** 10.1016/j.colsurfb.2016.08.044

**Published:** 2016-12-01

**Authors:** Roser Sabater i Serra, Laia León-Boigues, Antonio Sánchez-Laosa, Luis Gómez-Estrada, José Luis Gómez Ribelles, Manuel Salmeron-Sanchez, Gloria Gallego Ferrer

**Affiliations:** a Centre for Biomaterials and Tissue Engineering, Universitat Politècnica de València, Spain; b Biomedical Research Networking Center in Bioengineering, Biomaterials and Nanomedicine (CIBER-BBN), Spain; c Ikasia Technologies SL, València, Spain; d Division of Biomedical Engineering, School of Engineering, University of Glasgow, United Kingdom

**Keywords:** Poly(ethyl acrylate), Crosslinked network, Scaffolds, Bioactive substrates, Fibronectin, 3D-fibrillogenesis

## Abstract

Poly(ethyl acrylate) (PEA) induces the formation of biomimetic fibronectin (FN) (nano)networks upon simple adsorption from solutions, a process referred to as *material-driven FN fibrillogenesis*. The ability of PEA to organize FN has been demonstrated in 2D and 2.5D environments, but not as yet in 3D scaffolds, which incorporate three-dimensionality and chemical crosslinkers that may influence its fibrillogenic potential. In this paper we show for the first time that while three-dimensionality does not interfere with PEA-induced FN fibrillogenesis, crosslinking does, and we determined the maximum amount of crosslinker that can be added to PEA to maintain FN fibrillogenesis. For this, we synthesised 2D substrates with different amounts of crosslinker (1–10% of ethylene glycol dimethacrylate) and studied the role of crosslinking in FN organization using AFM. The glass transition temperature was seen to increase with crosslinking density and, accordingly, polymer segmental mobility was reduced. The organization of FN after adsorption (formation of FN fibrils) and the availability of the FN cell-binding domain were found to be dependent on crosslinking density. Surface mobility was identified as a key parameter for FN supramolecular o**r**ganization. PEA networks with up to 2% crosslinker organize the FN in a similar way to non-crosslinked PEA. Scaffolds prepared with 2% crosslinker also had FN (nano)networks assembled on their walls, showing PEA’s ability to induce FN fibrillogenesis in 3D environments as long as the amounts of crosslinker is low enough.

## Highlights

• Poly(ethyl acrylate) crosslinking alters adsorbed fibronectin (FN) organization.
• FN fibrillogenesis induced by poly(ethyl acrylate) is kept up to 2% of crosslinker.
• Adsorbed FN on scaffolds showed physiological-like nano(networks).
• FN fibrillogenesis induced by poly(ethyl acrylate) was proved in 3D environments.

## Graphical abstract

**Figure:**
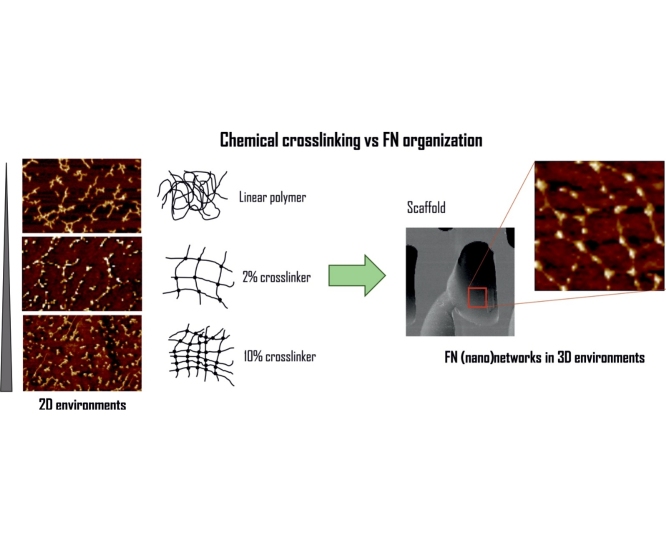

## 1 Introduction

Biomaterials play a key role in regenerative medicine, acting as synthetic extracellular matrices (ECM). Exogenous ECM is designed to bring cells into contact in a physiological-like three-dimensional (3D) environment, providing the necessary support for cell growth, differentiation and production of a natural ECM [1], [2], [3]. The main function of scaffolds or 3D engineered ECM is to mimic the functions of natural ECM, acting as a support to allow tissue development, control tissue structure and regulate the cell phenotype [4], [5], [6], [7].

However, these synthetic biomaterials are biologically inert and have to be functionalised with adhesive proteins or active biomolecules to become bioactive, so that the material becomes biologically active and it is recognized by the cells, enabling adhesion, proliferation and differentiation [8], [9], [10], [11], [12], [13]. One of the common strategies used to enhance bioactivity is the adsorption of adhesive proteins, such as fibronectin (FN), laminin and fibrinogen, on the material surface [12], [13]. The composition of the adsorbed protein layer is a key factor in cell behaviour, as the cells depend on specific proteins for anchorage and extracellular instructions. The required protein, properly presented, can thus stimulate an effective cell response and promote tissue regeneration [14]. Proteins are adsorbed in different quantities, densities, conformations, and orientations, according to the physico-chemical properties of the substrate [15], [16], [17], [18], [19], [20], [21], [22].

Previous work has shown that certain materials, such as poly(alkyl acrylates) with a vinyl backbone and side groups —CO_2_(CH_2_)_x_H, where x = 2, 4, and 6, are able to biomimetically induce the organization of FN, a process referred to as *material-driven fibronectin fibrillogenesis*. As consequence of protein-material interactions, a physiological-like FN (nano)network is organised upon simple adsorption of FN from a protein solution [23], [24]. Poly(ethyl acrylate) substrates (PEA) with a —CO_2_(CH_2_)_2_H side chain promote highly interconnected FN fibrils that have been shown to be biologically active in terms of cell adhesion, signalling, matrix reorganization and cell differentiation [13], [25], [26], [27], [28], [29]. The FN (nano)network induced by PEA substrates enhances cell adhesion with a higher number of actin stress fibres and focal adhesion kinase activity. Furthermore, FN provides greater exposure of cell binding domains (integrin binding regions), which translates into higher cell differentiation [26], [27].

The way in which cells interact with synthetic scaffolds is determined by the functional properties that the scaffold can achieve, and this, in turn is determined by material chemistry and manufacturing conditions. Transport requirements for cell nutrition, porous channels for cell migration, and surface characteristics for cell attachment will be the specific parameters for the engineered 3D structures [30].

In this study, we engineered bioactive PEA-based scaffolds for tissue engineering. PEA scaffolds with interconnected pores larger than 300 μm for efficient nutrient and metabolite transport [31], [32] were synthesised and coated with FN to enhance bioactivity. The scaffolds were produced by polymerization in the presence of a porogen template and a subsequent particle leaching method. This process required polymer crosslinking to allow several cycles of swelling-shrinkage to remove all traces of porogen. 2D substrates were prepared with different amounts of crosslinker and subsequently coated with FN in order to study the organization and conformation of the protein adsorbed.

The optimal amount of crosslinker for scaffold manufacturing was selected from 2D substrates that induced FN (nano)fibril formation in the same way as the non-crosslinked ones. The scaffold’s morphology and the organization of the FN adsorbed on the scaffold pores were then analysed.

The main contribution of this work is that it shows that PEA-based 3D scaffolds sustain the organization of the fibronectin in their pores into physiological-like (nano)networks in the same way as in 2D substrates and fibres, the so-called 2.5D environments [13], [23], [24], [25], [26], [27], [33].

## 2 Materials and methods

### 2.1 Synthesis of 2D substrates with different amounts of crosslinker

A series of poly(ethyl acrylate) polymer films were obtained by radical polymerization. Briefly, ethyl acrylate (99%, Sigma-Aldrich) monomer was mixed with 0.5 wt.% benzoin (98% pure Scharlab) as photoinitiator and different proportions (0, 1, 2, 3, 5 and 10 wt.%) of ethylene glycol dimethacrylate (EGDMA) (98%, Sigma-Aldrich) as crosslinker. The reaction was carried out in ultraviolet light for 24 h. After polymerization, samples were washed with ethanol in a Soxhlet extractor for 24 h in order to remove low molecular weight substances, except the sample with 0% of EGDMA, which was dried in a vacuum to constant weight. The 2D substrates will be identified hereinafter as PEA-X%, in which X is the percentage of EGDMA. The films obtained were approximately 1 mm thick.

### 2.2 Preparation of the scaffolds

Scaffolds with channels aligned in orthogonal directions were prepared by radical polymerization of the monomer mixture inside a template that was subsequently removed by leaching. Commercially available poly(vinyl alcohol) water soluble polymer (PVA) (Plastic2print) was used to prepare the sacrificial template by 3D-printing. The template was prepared with Ikasia Technologies SL software, it was circular in shape (diameter 50 mm x 5 mm thick) and formed by stacked layers of 400 μm diameter PVA threads. The distance between two threads was 300 μm and the orientation of the threads in each layer was perpendicular to the adjacent one. Ethyl acrylate monomer was then mixed with 1 wt.% of benzoyl peroxide (BPO) (97%, Fluka) as thermal initiator and 2 wt.% of EGDMA as crosslinker, stirred for 15 min, injected into the PVA template and polymerized for 24 h at 60 °C. In order to avoid the evaporation of the monomer solution during polymerization, the base and external walls were sealed with a thick impermeable PVA layer and the upper part was sealed by a glass. After polymerization, the PVA template was dissolved in water for 3 days at 80 °C. Thereafter, the PEA scaffolds were first swollen in ethanol (98%, Scharlab) and immersed in water at 80 °C for 24 h. This process was repeated three times to fully eliminate any trace of porogen. Finally, the scaffolds were dried under vacuum at room temperature. The scaffold thickness was approximately 4 mm.

### 2.3 Differential scanning calorimetry (DSC)

DSC was carried out on a Metter Toledo 823e on ca. 5 mg samples. Nitrogen gas was purged through the DSC cell at a flow rate of 29 mL/min. After erasing the effects of any previous thermal history by heating to 150 °C, samples of PEA-X% films were subjected to a cooling scan down to −100 °C followed by a heating scan up to 100 °C, both at a rate of 10 °C/min.

The glass transition temperature, *T_g_*, was obtained from the cooling scan as the midpoint of the change in specific heat capacity, *c_p_*. The width of the glass transition, *ΔT_g_*, was obtained by the intersections of the tangent line at the inflexion point with the extrapolated glass and liquid lines. Likewise, the specific heat capacity increment at the glass transition, *Δc_p_(T_g_)*, was determined as the difference in heat capacity between extrapolated liquid and glass lines at *T_g_*.

### 2.4 Dynamic-mechanical analysis (DMA)

Dynamic mechanical analysis was performed on 2D PEA-X% substrates on a DMA 8000 (Perking Elmer) at a frequency of 1 Hz in the tension mode. The temperature dependence of the storage and loss modulus, *E*’ and *E*”, respectively, were measured in the temperature range −50 to 80 °C at a rate of 2 °C/min. The samples for these experiments were rectangular (20 mm × 6 mm) and ca. 1 mm-thick.

### 2.5 Swelling experiments

The swelling degree of PEA-X% networks (X = 1 to 10) was obtained gravimetrically. Dry discs (5 mm diameter) were cut from polymerized sheets. Swelling experiments were performed by immersing the samples in ethanol until equilibrium at room temperature. The swelling degree was expressed as the amount of solvent per unit mass of the dry polymer.

### 2.6 Thermogravimetric analysis (TGA)

Measurements were performed on a Metter Toledo TGA/DSC 2 Star System. 5–10 mg weight samples were placed on the balance and the temperature was raised from 30 to 800 °C at a heating rate of 10 °C/min. The mass of the sample was monitored as a function of temperature in PEA-X% films and PEA scaffolds.

### 2.7 Water contact angle

Water drop contact angles of PEA-X% substrates were measured by a Dataphysics OCA. The volume of the drop was 8 μL and the measurement was performed after 30 s of substrate-water contact. The experiment was replicated five times for each sample.

### 2.8 Morphological characterization of PEA-based scaffolds

Scaffold morphology was observed with a scanning electron microscope (SEM, JSM-6300, JEOL). Samples were mounted on copper stubs and gold coated using a sputter coater (Polaron, SC502). The working distance was fixed at 15 mm and acceleration voltage at 13 kV.

### 2.9 Density and porosity measurements

A Mettler Toledo analytical balance AE 240 balance with a sensitivity of 0.01 mg and a ME 33360 accessory kit was used to obtain the density of PEA substrates and open porosity of scaffolds in accordance with European Standard EN 993-1.

The density of PEA-X% films was determined by applying the buoyancy method as:

(1) $$\rho_{PEA-X\%}=\frac{m_{\text{air}}}{m_{air}-m_{n-oc\tan e}}\rho_{n-oc\tan e}$$

where *m_air_* is the weight of the sample in air, *m_n-octane_* is the weight of the sample immersed in *n*-octane and *ρ_n-octane_* is the density of *n*-octane (0.703 g/cm^3^). N-octane was chosen because it is not a solvent of the polymer and has lower density than the materials.

In order to obtain the scaffold porosity, different samples were weighed in three conditions: *i*) in air (*m_Scf-air_*), *ii*) weighed in air (*m_Scf-filled-air_*) with the pores filled with *n*-octane (under vacuum) and *iii*) immersed in *n*-octane (*m_Scf-filled-immersed_*) with the pores filled with *n*-octane. The open porosity π*_a_* in volume percent was calculated as follows:

(2) $$\pi_{a}=\frac{m_{Scf-filled-air}-m_{Scf-air}}{m_{Scf-filled-air}-m_{Scf-filled-inmersed}}\cdot 100$$

Five measurements were carried out and the values were averaged.

### 2.10 FN organization on material surfaces. Atomic force microscope (AFM) experiments

AFM was performed in a NanoScope IIIa controller from Veeco operating in tapping mode in air. Nanoscope 5.30r2 software was used for image processing and analysis. Si-cantilevers (Veeco) were used with a resonance frequency of 75 kHz and force constant of 2.8 N/m. The phase signal was set to zero at a frequency 5–10% lower than the resonance frequency. Drive amplitude was 600 mV and the amplitude set point Asp was 1.8 V. The ratio between the amplitude set point and the free amplitude A∞/A0 was kept equal to 0.7.

To analyse FN organization on material surfaces (both PEA-X% films and PEA-based scaffolds), the samples were covered with a 10 μg/mL solution for 10 min (FN from human plasma, Sigma-Aldrich) and Milli-Q water. After adsorption, the samples were rinsed with Milli-Q water to eliminate the non adsorbed protein.

### 2.11 Adsorbed FN quantification

Human plasma FN was adsorbed from a 20 μg/mL solution in Dulbecco's phosphate-buffered saline (DPBS) on 10 mm diameter PEA-X% substrates until complete saturation (1 h) at room temperature. A single drop of the FN solution (200 μL) was deposited and spread to cover the whole surface of the substrate. Afterward, the supernatant was collected and quantified by a Micro BCA Protein Assay Kit (Thermo Scientific). The amount of adsorbed FN was obtained as the difference between the amount of FN in the initial solution and in the supernatant.

### 2.12 FN conformation (availability of the cell binding domain of FN)

The relationship between the availability of the cell-binding domain of FN and the degree of fibrillogenesis on the material surface was investigated by means of an enzyme-linked immunosorbent assay (ELISA). FN was adsorbed from a 20 μg/mL solution in DPBS for 1 h on 10 mm PEA-X% films. The samples were then rinsed with DPBS to eliminate the non-adsorbed protein. After adsorption, the samples were blocked in 1% BSA/DPBS and incubated with primary antibody Anti-Fibronectin cell binding region (1:500 MAB1937, Merck-Millipore) in blocking solution (1 h at 37 °C), rinsed in 0.5% Tween-20/DPBS and incubated with alkaline phosphatase conjugated secondary antibody (1:5000) for 1 h at 37 °C, followed by incubation with 4-methylumbelliferyl phosphate substrate (Sigma-Aldrich) for 45 min at 37 °C. A fluorescence plate reader (Victor III, PerkinElmer) at 365 nm/465 nm was used to quantify the reaction products.

### 2.13 Statistics

All the experiments were performed in triplicate unless otherwise noted. Data were reported as mean – standard error. Where relevant, one-way ANOVA (GraphPad Prism 6.0 software) was used for statistical analysis; a 95% confidence level was considered significant (p < 0.05).

## 3 Results and discussion

### 3.1 Polymer networks physico-chemical properties

**Fig. 1:**
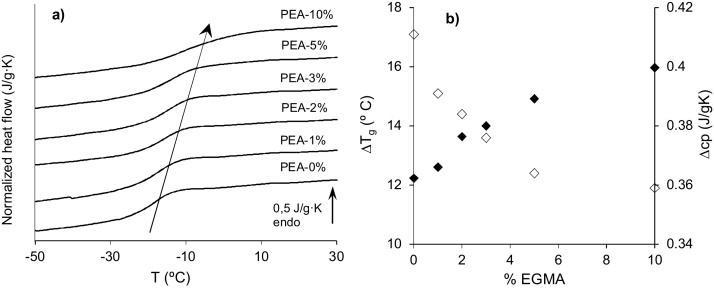
DSC measurements in 2D substrates. a) Normalized heat flow on cooling at 10 °C/min. b) (♦) width of glass transition, *ΔT_g_*_,_ and (◊) specific heat capacity increment at glass transition, *Δc_p_*, as function of%EGDMA.

Fig. 1a shows the cooling DSC thermograms for the 2D substrates prepared with varying amounts of crosslinker. The glass transition temperature, *T_g_*, shifts to higher temperatures as the percentage of EGDMA crosslinker is increased (Fig. 1a and Table 1). EGDMA has two double bonds that open in the presence of a catalyser forming four radicals that can covalently bond four polymeric PEA chains per EGDMA molecule, imposing a restriction on the molecular mobility of the polymer. Chemical crosslinking is an effective way of preventing polymer dissolution, thus allowing porogen washing with a good solvent of the polymer. Since the glass transition temperature, *T_g_*, represents the onset of cooperative segmental motions, increasing crosslink density reduces long-range chain movements. *T_g_* rises and more energy is required to induce segmental motions [34]. The specific heat capacity increment at glass transition, *Δc_p_*, decreases from 0.41 J/g K for PEA without crosslinker to 0.36 J/g K for PEA-10% (Fig. 1b), denoting that molecular mobility diminishes as the network becomes more crosslinked [35], [36], [37], [38]. The width of the glass transition, *ΔT_g_*, linked to the distribution of mobility of the polymer segments, raises as the amount of crosslinker increases, suggesting a structural inhomogeneity in the network that may be attributed to the existence of nanodomains with different mobility (Fig. 1b). [35], [38].

The elastic modulus, *E*’, obtained from the DMA measurements at 25 °C is depicted in Table 1. It can be observed that the value rises with increased amounts of EGDMA, from 0.68 MPa for PEA-0% until 4.32 MPa for PEA-10%. Polymer crosslinking has strong effects on the mechanical properties of the material, particularly in elastomers, where raising crosslinking density leads to an increase of the elastic modulus, as predicted by the rubber elasticity theory [39]. The Young’s modulus obtained in the substrates with 2% EGDMA is 16% higher than the one obtained for non-crosslinked PEA. PEA-10% shows values more than 5 times higher, although the samples with this composition became brittle. This change in mechanical properties has no significant influence on the surface wettability. All PEA-X% substrates maintain high contact angle values, showing hydrophobic behaviour (Table 1), with no substantial differences in wettability, regardless of the amount of crosslinker.

Experimental results can provide microscopic parameters of PEA network structure and morphology that can throw light on the influence of crosslinkers on the PEA’s capacity to induce FN fibrillogenesis. Stoichiometry provides the mean number of monomeric units between crosslinks, *ν_st_*, which in an ideal network with tetrafunctional crosslinks (due to EGDMA crosslinker) can be calculated as:

(3) $$\upsilon_{st}=\frac{m_{EA}\cdot M_{EGDMA}}{2m_{EGDMA}\cdot M_{EA}}$$

where *m_EA_* and *M_EA_* are the mass and molecular mass of the monomer, respectively, and m_EGDMA_ and *M_EGDMA_* are the same magnitudes of the crosslinker. For PEA-X% networks (*M_EA_* = 100 g/mol and *M_EGDMA_* = 198 g/mol), the values diminish as the amount of crosslinker increases from *ν_st_* = 98 for PEA-1% to *ν_st_* = 10 for PEA-10%, as depicted in Table 2.

Experimentally, this parameter can also be obtained from the rubber elasticity theory for the affine network [40] by calculating the mean molecular mass between crosslinks, ${\bar{M}}_{c}$, as follows:

(4) $${\bar{M}}_{c}=\frac{\rho_{PEA-X\%}3RT}{E'}$$

where *ρ_PEA-X%_* is the density of the network (Table 2), *E*’ is the elastic modulus in the rubbery region (Table 1), *R* is the universal gas constant and *T* is the temperature (298 K). The mean number of monomeric units per elastically-active chain (between crosslinks), *ν_el_,* can now be obtained as the quotient between the mean molecular mass (between crosslinks) and the molecular mass of the EA monomer:

(5) $$\nu_{el}=\frac{{\bar{M}}_{c}}{M_{EA}}$$

The values for these effective monomeric units, **ν*_el_*, follow the same trend as those obtained for the ideal network, **ν*_st_,* and are in the same order of magnitude, ranging from 128 for PEA-1% to 20 for PEA-10% [41]. Regardless of the amount of crosslinker, the values of **ν*_el_* are higher than **ν*_st_* for all substrates. This is likely due to the presence of network defects, such as crosslinker molecules lost in inelastic junction, crosslinker units at the end of loose chains or consecutively linked crosslinker units that are not part of the effective network. [41], [42], [43], [44]. The mesh size of the network, *ξ*, which characterizes the mean distance between junctions of the network, can be obtained from the mean number of effective monomeric units between crosslinks, **ν*_el_*, as:

(6) $$\xi =C^{\frac{1}{2}}n_{el}^{\frac{1}{2}}l$$

where *C* is the characteristic ratio for the polymer and *l* is the length of the chain unit. This expression is valid when the number of units of the chain is above 10 (in our networks the values vary between 20 and 128). Considering *C* = 6 [45] and 1.58 Å as the length for vinyl chains [46], the mesh size ranges between 4.38 nm for the network with 1% crosslinker to 1.72 nm for the network with the highest amount of crosslinker (Table 2).

The crosslinking density of the networks, defined as the molar concentration of elastically effective chains per unit volume of polymer, *n_c_/V_pol_*, can be obtained as the quotient of the density of the network and the mean molecular mass between crosslinks:

(7) $$\frac{n_{c}}{V_{pol}}=\frac{\rho_{PEA-X\%}}{{\bar{M}}_{c}}$$

As expected, the crosslinking density of the network increases with the amount of crosslinker; the values rise from 8.85 × 10^−5^ mol/cm^3^ for PEA-1% to 5.82 × 10^−4^ mol/cm^3^ for the network with 10% EGDMA (Table 2).

In order to determine the swelling degree of PEA-X% networks, *w*, obtained as the amount of solvent per unit mass of the dry network, the samples were immersed in ethanol, the solvent later used to wash the scaffolds to remove any traces of porogen (Table 2). The swelling degree was considerably reduced as the amount of crosslinker was increased, going from ca. 71% for PEA-1% to 34.5% for samples with 10% of EGDMA. The Flory-Huggins interaction parameter between the polymer network and the solvent (ethanol), *χ_EtOH-pol_*, can be obtained from the Flory-Rehner Equation [47]:

(8) 0=ln(1−ϕpol)+ϕpol+χEtOH−polϕpol+vsncVpolϕpol13

where *ϕ_pol_* is the volume fraction of polymer in the swollen network (solvent content in equilibrium), *v_s_* is the molar volume of the solvent and *n_c_/V_pol_* is the previously calculated crosslinking density. We found that the interaction parameter, *χ_EtOH-pol_*_,_ increases slightly with the crosslinking density (Table 2), indicating that the parameter is affected by the chemical modifications involved in crosslinking, as has been reported elsewhere [48], [49], [50]. This could be due to a possible ‘copolymer’ effect between the EA monomer and the crosslinker, in which the final network has a copolymer structure, which consists of joined EA and EGDMA units between the network junctions, together with the network junctions themselves [49]. EGDMA units, copolymerized with EA units but not forming part of the junctions, are probably the cause of having less crosslinked networks than ideally predicted (**ν*_el_* higher than **ν*_st_*). This can also be related to the results obtained from DSC experiments, which point toward structural inhomogeneities in the networks, related to nanodomains with different segmental mobility.

### 3.2 FN organization on 2D material surfaces

**Fig. 2:**
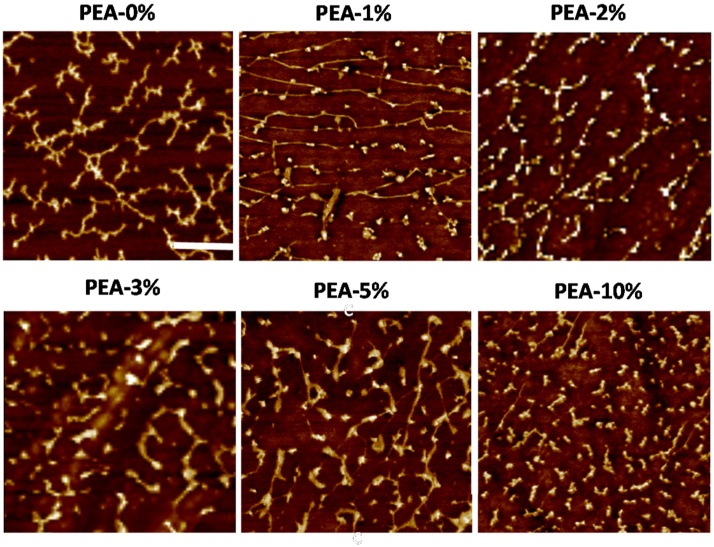
AFM images (phase magnitude) of FN adsorbed on 2D substrates from solutions with a concentration of 10 μg/mL (t_adsorption_: 10 min). The scale bar is 0.25 μm.

The AFM images in Fig. 2 show the FN distribution on PEA-X% substrates after adsorption FN from 10 μg/mL solutions. The concentration usually employed when coating a substrate for cell culture purposes is 20 μg/mL [51], [52], [53], however, in this work we used 10 μg/mL with the aim of studying the first stages in the FN (nano)network formation by the development and subsequent interconnection of fibrils. Different protein organizations were found to vary with the amount of EGDMA in the monomer mixture. The non-crosslinked PEA-0% substrates show highly extended FN fibrils uniformly distributed on the surface. As the amount of crosslinker increases, the FN fibrils display a more rounded structure with shorter elongations. Substrates with 1 and 2% EGDMA are able to organize the FN in a similar way as non-crosslinked PEA, with partially interconnected extended fibrils. In substrates with more than 2% EGDMA, the fibrils are shorter and less extended and are organised in a more globular conformation. This is particularly noticeable in PEA-10% substrates, in which FN fibril formation is rarely observed. Only small aggregates can be seen on the surface, without the usual elongations that lead to the formation of the FN (nano)network. All the substrates show similar wettability values (Table 1), suggesting that surface chemistry is not a relevant parameter in FN organization.

These results suggest a close dependence between the mobility of polymer chains and the FN organization on the surface [23], [27]. When the amount of crosslinker increases, *T_g_* rises monotonically, reducing surface mobility. As a result, the formation of extended FN fibrils is hindered in substrates with diminished mobility. Glass transition was reduced by between 5 and 10 °C in substrates with ≥3% EGDMA, which are those with the less interconnected FN fibrils.

FN adsorbed on a series of copolymers with different ethyl acrylate/methyl acrylate (EA/MA) ratios showed significant changes in FN organization and conformation after adsorption [27]. In the copolymers, *T_g_* increased monotonically with the number of MA units, i.e. the molecular mobility of the system decreased from PEA (EA/MA 100/0) to PMA (EA/MA 0/100). Fully interconnected FN networks were only obtained with high EA/MA ratios and the FN fibrils became less interconnected with increasing MA units. Our results are in good agreement with those obtained with EA/MA copolymers, in which EA/MA 70/30 copolymer, with less segmental mobility (*T_g_* ca. 12 °C higher than pure PEA), showed diminished FN organization after adsorption. Further reduction in surface mobility (adding MA units) resulted in less extended and interconnected FN fibrils. Differentiation of murine myoblasts was enhanced on the copolymers with more interconnected FN fibrils.

In a parallel way, previous studies on a family of polyalkyl acrylates with an increasing length of the side group —CO_2_(CH_2_)_x_H (x = 1, 2, 4 and 6) have shown the organization of adsorbed FN on substrates with different mobility [23], [24], [29]. For this family of polymers, *T_g_* decreased monotonically as the length of the side group increased. Substrates with the highest *T_g_*, and therefore the lowest segmental mobility (x = 1), induced globular FN organization. In contrast, substrates with higher segmental mobility (x ≥ 2), supported the formation of fibrillar protein (nano)networks that reflected the mobility of the underlying polymer surface. This change of FN organization from globular to fibrillar is thought to be driven by the orientation of key hydrophobic residues to interact with the polymer backbone [13], [23].

#### 3.2.1 Availability of FN domains

**Fig. 3:**
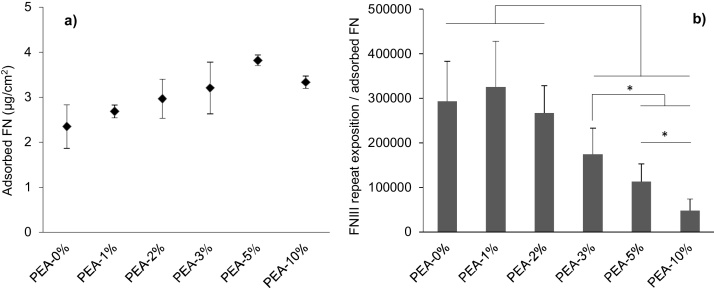
Fibronectin conformation on PEA 2D substrates. a) Surface density of adsorbed FN. b) Relationship between the exposure of FNIII_8_ repeat obtained by enzyme-linked immunosorbent assay and the amount of crosslinker. (FN concentration: 20 μg/mL, t_adsorption_: 1 h).

The surface density of FN on the different PEA-X% substrates, quantified by the BCA assay, shows similar levels of adsorbed protein (Fig. 3a). However, the availability of the cell-binding domain of FN on the different substrates (mAB1937 binds FNIII_8_ next to FNIII_9,_ where the RGD sequence is located [27], [54]) was found to depend on the percentage of crosslinker (Fig. 3b). The different FN organization on the surface involves different conformation of the protein, as indicated by the increased availability of the cell-binding domain in substrates with extended fibrils (Fig. 3b). Substrates with 1 and 2% EGDMA show no significant differences in the exposure of the cell-binding domain (FNIII_8_ repeat) as compared to linear PEA. However, for higher amounts of crosslinker (3, 5 and 10% EGDMA), the exposure of this specific domain is substantially reduced. These results, with 20 μg/mL FN concentration (the usual concentration used in cell culture), indicate the relationship between FN organization on the material surface and the conformation of the layer of protein adsorbed. The correlation between FN organization and cell behaviour has been shown in [55], [56]. A high degree of FN fibrillogenesis leads to better cell adhesion, with a higher number of actin fibres and more efficient cell signalling. This means that proliferation and cell differentiation are enhanced by the higher cell signalling efficiency [26], [27], [28], [33], [51], [55], [56], [57], [58].

### 3.3 Scaffolds morphology and physico-chemical properties

In order to prepare PEA-based scaffolds, we have to consider the factors outlined for both good processing conditions (scaffold manufacturing) and the ability to promote FN fibrillogenesis (which implies high exposure of the cell-binding domain). From the point of view of processing conditions, the removal of all traces of PVA porogen requires several cycles of washing with water, followed by the swelling of the polymer. In this process, the degree of swelling must be limited in order to avoid the collapse of pores during drying (Table 2). Polymerization with >1% EGDMA and subsequent swelling in ethanol were considered suitable. However, FN organization and conformation on the substrates after adsorption show that fibril formation and cell domain exposure fall as the% of crosslinker increases. As only substrates with 1 and 2% EGDMA have similar behaviour to linear PEA (are able to promote the FN fibrillar (nano)network and give good exposure of the cell binding domain), we chose 2% EGDMA as optimal for scaffold manufacturing. It is worth noting that this composition shows an increase of ca. 20% in the elastic modulus, *E*’ (Table 1), a characteristic required for strengthening the scaffold structure.

**Fig. 4:**
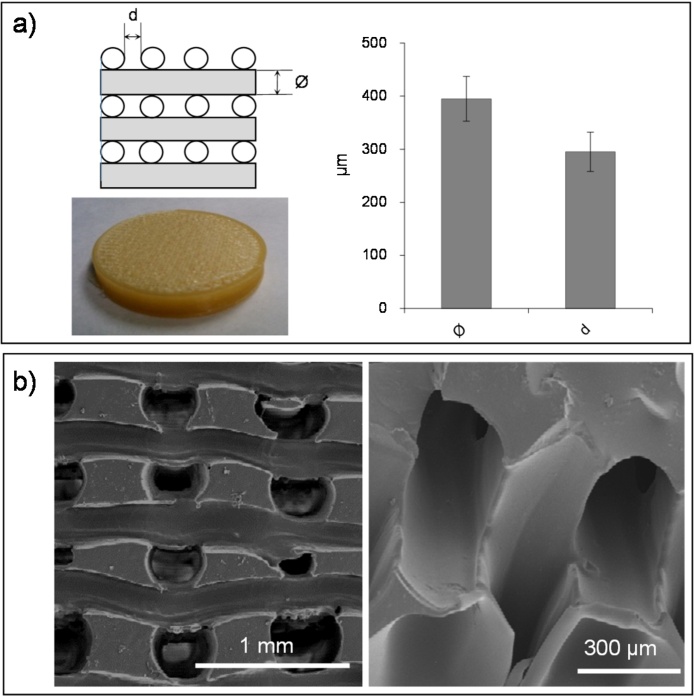
a) PVA sacrificial template by 3D-printing (cross-section structure and top view) and diameter, ϕ, and distance, d, between PVA fibres. b) SEM images of PEA-based scaffolds (2% of EGDMA) with channelled interconnected pores (cross-section).

The dimensions of PEA scaffolds prepared by a combination of radical polymerization and particle leaching with 2% of EGDMA as crosslinker were 5 cm in diameter and ca. 4 mm thick. Fig. 4a shows the dimensions of the sacrificial template prepared by 3D printing (Fig. 4a) and the final structure of the PEA scaffold (Fig. 4b). They showed a homogeneous structure with interconnected pore channels oriented in orthogonal directions, as can be seen in the SEM images (Fig. 4b), with an average porosity of 52.2%. The mean pore diameter is 317 ± 13 μm while the trabeculae thickness is 370 ± 26 μm. The pore walls have a smooth surface, with no traces of residual PVA fibres. Open and interconnected pores are essential for tissue vascularization and the formation of new tissues. It has been reported that pores greater than 300 μm facilitate vascularization [31], [32]. If the pores become too large, the scaffolds mechanical properties can be compromised by the void volume, and also (in highly porous scaffolds) the specific surface would be reduced and cell adhesion limited [59].

**Fig. 5:**
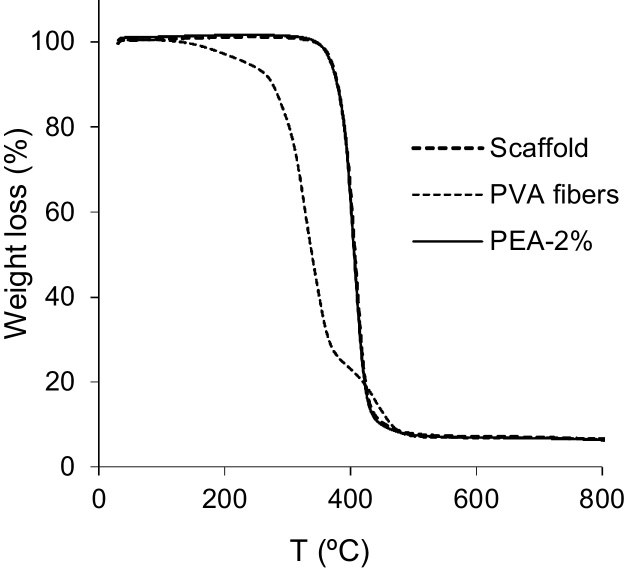
Thermogravimetry of PEA-2% network, PEA-based scaffold (2% of EGDMA) and PVA fibres.

Additional thermogravimetry analyses were performed on the scaffolds, 2D substrates with the same percentage of crosslinker (PEA-2%) and PVA fibres in order to discard any remaining PVA fibres on the scaffold walls. The results indicate that these fibres had been completely removed, as no traces were found in the TGA scan in which both scaffold and PEA-2% curves overlap (Fig. 5).

### 3.4 FN organization on 3D substrates (PEA scaffolds)

**Fig. 6:**
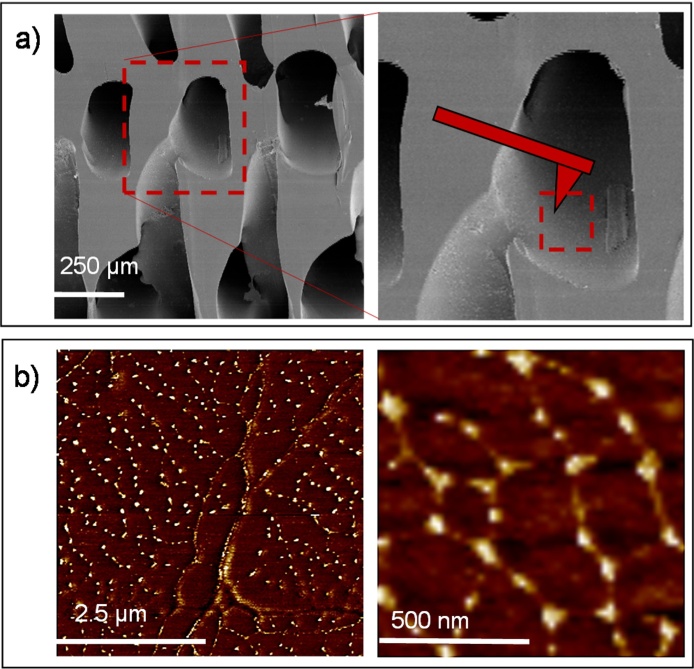
a) PEA-based scaffold (cross-section, SEM images) with a sketch showing the AFM probe within the pore (tapping mode). b) AFM images (phase magnitude) of FN adsorbed on the scaffold pores from a solution with 10 μg/mL of concentration (t_adsorption_: 10 min).

PEA scaffolds were coated with FN (from a solution of 10 μg/ML) in order to study the formation of FN (nano)fibrils in 3D substrates, as shown previously in 2D and 2.5D environments [13], [24], [25], [26], [27], [33]. As expected, AFM images (Fig. 6b) show a fibrillar organization of the FN absorbed on the scaffold pore walls, as was found in 2D substrates with the same amount of crosslinker (PEA-2% in Fig. 2). Extended FN fibrils with a network-like structure can be observed, showing the first steps of fibrillogenesis induced by PEA-based substrates in 3D environments.

## 4 Conclusions

In this work we engineered PEA-based 3D environments able to promote the organization of fibronectin into physiological-like (nano)networks. Tissue engineering scaffolds were prepared by a template leaching technique (PVA fibres) followed by radical polymerization. Polymer crosslinking was required to remove PVA fibres after PEA polymerisation, as this process involves the immersion of the samples in a solvent that also dissolves PEA. 2D substrates prepared with varying amounts of crosslinker (EGDMA) showed significant influence of crosslinking density on the organization and conformation of FN. 2D substrates with up to 2% crosslinker behaved similarly to non-crosslinked ones, which suggests that surface mobility is a key parameter in leading FN organization. Scaffolds with pore size >300 μm, highly interconnected pores and approximately 50% porosity were prepared using 2% of crosslinker. FN absorbed on the scaffold walls displayed a fibrillar organization into nanonetworks, demonstrating the ability of PEA to induce FN fibrillogenesis in 3D environments.

## Figures and Tables

**Table 1:** Experimental glass transition (*T_g_*), Elastic Modulus (*E′)*, at 25 °C and static water contact angle on 2D substrates.

Sample	T_g_ (°C)	E' (MPa)	Static contact angle (°)
PEA-0%	−20.1 ± 0.8	0.68 ± 0.06	91 ± 2
PEA-1%	−18.0 ± 0.8	0.66 ± 0.10	96 ± 3
PEA-2%	−17.1 ± 0.5	0.79 ± 0.05	93 ± 3
PEA-3%	−16.4 ± 0.8	1.01 ± 0.07	89 ± 3
PEA-5%	−15.1 ± 0.9	1.63 ± 0.10	91 ± 1
PEA-10%	−9.0 ± 1.0	4.32 ± 0.07	89 ± 2

**Table 2:** Density of 2D substrates (*ρ_PEA-X%_*), mean molecular mass between crosslinks, mean number of units between crosslinks obtained from the ideal network (**ν*_st_*), mean number of monomer units per elastically active chains *(*ν*_el_*), crosslinking density (n_c_/V_pol_), swelling degree in ethanol (*w*), interaction parameter between polymer network-solvent (*χ_EtOH-pol_*), mesh size of the network (*ξ*).

%EGDMA	1%	2%	3%	5%	10%
*ρ_PEA-X%_* (g/cm^3^)	1.132	1.135	1.136	1.143	1.151
${\bar{M}}_{c}$ (g/mol)	12801	10826	8367	5201	1976
**ν*_st_*	98	49	33	20	10
**ν*_el_*	128	108	84	52	20
n_c_/V_pol_ (mol/cm^3^)	8.85E-05	1.05E-04	1.36E-04	2.20E-04	5.82E-04
*w* (%)	70.98	62.49	57.15	48.08	34.48
*χ_EtOH-pol_*	0.75	0.78	0.80	0.83	0.90
*ξ* (nm)	4.38	4.03	3.54	2.79	1.72
